# Adaptive Robust Trajectory Tracking Control of Multiple Quad-Rotor UAVs with Parametric Uncertainties and Disturbances

**DOI:** 10.3390/s21072401

**Published:** 2021-03-31

**Authors:** Yasir Mehmood, Jawad Aslam, Nasim Ullah, Md. Shahariar Chowdhury, Kuaanan Techato, Ali Nasser Alzaed

**Affiliations:** 1 School of Mechanical and Manufacturing Engineering, National University of Science and Technology, Islamabad 44000, Pakistan; ymahmood.me19smme@student.nust.edu.pk (Y.M.); jawadaslam@smme.nust.edu.pk (J.A.); 2 Department of Electrical Engineering, College of Engineering Taif University, Al-Hawiyah, Taif P.O. Box 888, Saudi Arabia; 3 Faculty of Environmental Management, Prince of Songkla University, Hat Yai 90112, Thailand; mdshahariar.c@psu.ac.th (M.S.C.); kuaanan.t@psu.ac.th (K.T.); 4 Environmental Assessment and Technology for Hazardous Waste Management Research Center, Faculty of Environmental Management, Prince of Songkla University, Hat Yai 90112, Thailand; 5 Department of Architecture Engineering, College of Engineering Taif University, Al-Hawiyah, Taif P.O. Box 888, Saudi Arabia; alzaed@tu.edu.sa

**Keywords:** quad-rotor control, adaptive robust control, super twisting sliding mode control, formation control

## Abstract

Recently, formation flying of multiple unmanned aerial vehicles (UAVs) found numerous applications in various areas such as surveillance, industrial automation and disaster management. The accuracy and reliability for performing group tasks by multiple UAVs is highly dependent on the applied control strategy. The formation and trajectories of multiple UAVs are governed by two separate controllers, namely formation and trajectory tracking controllers respectively. In presence of environmental effects, disturbances due to wind and parametric uncertainties, the controller design process is a challenging task. This article proposes a robust adaptive formation and trajectory tacking control of multiple quad-rotor UAVs using super twisting sliding mode control method. In the proposed design, Lyapunov function-based adaptive disturbance estimators are used to compensate for the effects of external disturbances and parametric uncertainties. The stability of the proposed controllers is guaranteed using Lyapunov theorems. Two variants of the control schemes, namely fixed gain super twisting SMC (STSMC) and adaptive super twisting SMC (ASTSMC) are tested using numerical simulations performed in MATLAB/Simulink. From the results presented, it is verified that in presence of disturbances, the proposed ASTSMC controller exhibits enhanced robustness as compared to the fixed gain STSMC.

## 1. Introduction

A flight in which more than one quad-rotors fly and maintain the relative distance among each other is called formation flight. Recently, the interest in the formation control of quad-rotors has attracted a lot of attention. This trend is due to its potential applications in the defense industry, aerial mapping, search and rescue operations, oil fields monitoring, agriculture and transportation of suspended loads [1]. It is expected that by 2027 the payload market value of global UAV may reach USD 3 billion due to its anticipated usefulness [2]. Multiple quad-rotors increase the capacity for equipping sensors, provides larger payload capacity and a wider surveillance area as compared to single quad-rotor [3,4,5,6,7]. However, controlling the formation of multiple quad-rotors in the presence of uncertainties is a challenging task. Moreover, the derivation of formation dynamic model for multiple UAVs in presence of external perturbations has also become an important topic. The transnational and rotational dynamics of a quad rotor is modeled as six degree of freedom nonlinear differential equations [8,9,10,11]. For multiple UAVs, different formation geometries exist depending upon the number of quad-rotors and the purpose of flight. These include V shape geometry and finger four geometry. V shape geometry is used in this paper for flight formation of three quad-rotors.The quad-rotors formation can be stepped up, stepped down, and leveled based on the altitude position between leader and follower. A leveled formation is used in this paper. Since a quad-rotor is a complex system with under-actuated multi variable non-linear model hence its formation control problem is more difficult to control. To ensure robust formation control, the modeling uncertainties and the disturbance due to wind gust must be compensated using appropriate control schemes.

To solve the formation control problem of the quad rotor, many research efforts have been made. In [12] the authors proposed a leader–follower formation control using classical proportional derivative scheme and fuzzy logic system for the formation pattern. However the above controller did not take uncertainties into considerations. In [13], a prescribed performance controller is proposed for formation control of multiple UAVs. The prescribed performance controller ensures robust formation pattern and trajectory. In [14], a classical PID control scheme with a sliding mode controller (SMC) is proposed for multiple quad-rotors. However the above mentioned PID-SMC controller ignores the disturbances and communication delays between multiple UAVs. In [15,16,17], classical SMC method is proposed for formation control problems of multiple UAVs, however the classical SMC method offers high frequency chattering in the excitation signal. Chattering phenomena degrades the life of the actuators.A control scheme for the circular formation scheme of multiple UAVs is presented in [18]. A classical PI control-based synchronization control for the formation of two UAVs is presented in [19]. In [20] a distributed controller is presented to compensate the communication delays in multiple UAVs formation. Similarly a nonlinear distributed controller is proposed for formation of micro UAVs [21]. In [22], the authors proposed a cohesive formation controller multiple UAVs.A back-stepping control scheme is proposed for the formation control of multiple UAVs [23]. Similarly a model predictive control scheme is proposed for multiple UAVs using adaptive gain tuning method in [24,25]. Velocity tracking and formation control of quad-rotors is achieved by the design of prior-bounded intermediary adaptive controller which gives the reference orientation and bounded control thrust [26]. A guidance algorithm based on Lyapunov function is used for the formation control of quad-rotors with attached slung load, where the quad-rotors are controlled using linear quadratic tracking controller [27]. Leader follower formation controller is designed for two parrot drones in [28], where a proportional derivative controller is implemented in the respective models. The problems related to formation and tracking control of quad-rotors in leader follower formation are addressed and a formation controller is designed to avoid collision in swarm [29]. Adaptive law for the formation control of swarm UAVs in leader follower mechanism influenced by motion constraints and and unknown external disturbances is discussed in [30]. For a swarm of three quad-rotors, a model reference adaptive control algorithm is presented in [31]. The controller gains are tuned online, by which the algorithm allows the system to adapt to unexpected disturbances. However, in this method, no robust controller is investigated. A semi physical platform for formation control of multiple fixed wings UAV is proposed in [32]. In [33], a detailed survey on low cost UAV platforms for infra structure monitoring is proposed.

The above cited work is specifically focused on the formation control of multiple UAVs. It is also necessary to describe the back ground of robust control system due to its utmost importance in control community. Robust control is designed for uncertain systems in which the uncertainty belongs to bounded set [34]. Robust controllers are designed both in frequency and time domains. A widely used frequency domain robust controller is the H${}_{\infty }$ method and it was first reported in [34]. Later on several variants of H${}_{\infty }$ control were reported in the literature such as loop shaping in [35], optimal H${}_{\infty }$ control using Riccati equations [36] and Linear Matrix Inequalities (LMIs)-based design in [37]. In frequency domain, the performance and stability of the control system is measured in terms of gain and phase margins, percent overshoot, rise, delay and settling times of the signals. Apart from frequency domain, a modern approach for designing robust controllers is the state space frame work. Sliding mode-based control (SMC) system variants are the most widely used methods and it find numerous applications in all areas of science and technology [38]. Classical SMC has several disadvantages such as high frequency chattering and asymptotic convergence property. These shortcoming are addressed by introducing new variants of SMC such as global sliding mode [39], LMI-based SMC [40], Higher order SMC [41,42], Lyapunov-based adaptive SMC and Non singular terminal SMC in [43]. A widely and important criteria to ensure the stability of the SMC controllers is the Luyapunov theorem [44]. The proposed theorems ensure global stability of the nonlinear systems and control using the condition that the disturbances are bounded.

Considering the aforementioned literature review, this paper proposes adaptive robust formation and trajectory tracking of multiple UAVs using super-twisting sliding mode control method.The proposed controller compensates for the disturbances using adaptive control laws derived by Lyapunov function method.System stability is ensured using Lyapunov theorem. Furthermore, the formation flight between multiple UAVs are also controller using super twisting sliding mode control methods.

The rest of the paper is organized into four parts. First part is related to “system description and mathematical modeling”. The objective of this part is the modelling of a single quad-rotor and transnational dynamics of multiple UAVs. The second part includes the trajectory and the formation controller formulation. Then the simulation results are presented and comparative analysis for different controllers is done. At last, conclusions are made. Following specific contributions are highlighted:

1. In presence of external disturbances, robust formation and trajectory tracking of multiple UAVs is achieved using adaptive super twisting sliding mode control method.
2. The adaptive laws are derived using Lyapunov theorem and implemented using projection operators.

## 2. System Description and Mathematical Modeling

**Figure 1:**
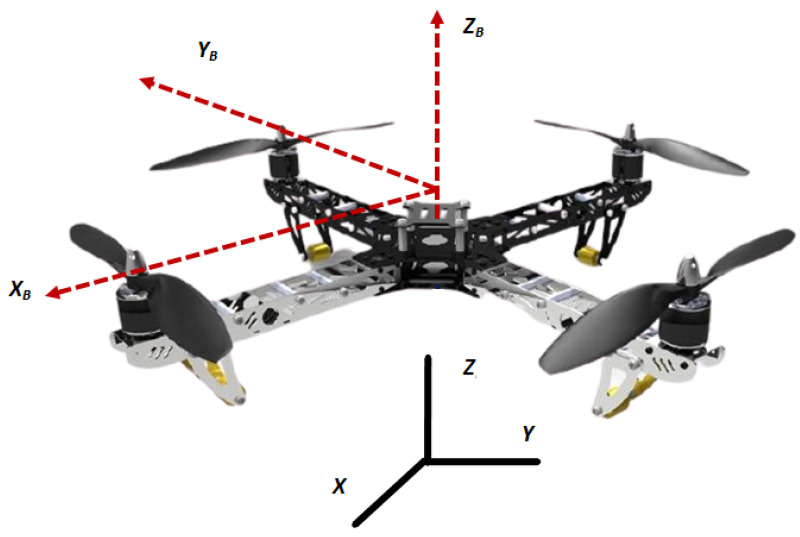
Quad-rotor in inertial reference frame.

Figure 1 shows a quad-rotor UAV in earth’s reference coordinates (X,Y,Z). Apart from inertial frame of reference, the body coordinates of the UAV are given as: ($X_{B}$$Y_{B}$$Z_{B}$). To derive the model, the following assumptions are made.

**Assumption** **1.** *It is assumed that the UAVs are represented by a symmetrical rigid body configurations with masses m.*

**Assumption** **2.** *The external disturbances affect the X and Y accelerations components of each UAV.*

**Assumption** **3.** *It is assumed that the disturbances are affecting the leader and followers UAV uniformly.*

(1) $$\begin{array}{c} \ddot{X_{i}}=\left(\sin\psi_{i}\sin\phi_{i}+\cos\psi_{i}\sin\theta_{i}\cos\phi_{i}\right)\frac{U_{1i}}{m_{Qi}}-D_{Xi} \end{array}$$

(2) $$\begin{array}{c} \ddot{Y_{i}}=\left(-\cos\psi_{i}\sin\phi_{i}+\sin\psi_{i}\sin\theta_{i}\cos\phi_{i}\right)\frac{U_{1i}}{m_{Qi}}-D_{Yi} \end{array}$$

(3) $$\begin{array}{c} \ddot{Z_{i}}=g-\left(\cos\theta_{i}\cos\phi_{i}\right)\frac{U_{1i}}{m_{Qi}}-D_{Zi} \end{array}$$

(4) $$\ddot{\phi_{i}}=\frac{I_{yi}-I_{zi}}{I_{xi}}\dot{\theta_{i}}\dot{\psi_{i}}-\frac{J_{ri}}{I_{xi}}\dot{\theta_{i}}\Omega_{ri}+\frac{l_{i}}{I_{xi}}U_{2i}-D_{\phi_{i}}$$

(5) $$\ddot{\theta_{i}}=\frac{I_{zi}-I_{xi}}{I_{yi}}\dot{\phi_{i}}\dot{\psi_{i}}-\frac{J_{ri}}{I_{yi}}\dot{\phi_{i}}\Omega_{ri}+\frac{l_{i}}{I_{yi}}U_{3i}-D_{\theta_{i}}$$

(6) $$\ddot{\psi_{i}}=\frac{I_{xi}-I_{yi}}{I_{zi}}\dot{\phi_{i}}\dot{\theta_{i}}+\frac{l_{i}}{I_{zi}}U_{4i}-D_{\psi_{i}}$$

**Figure 2:**
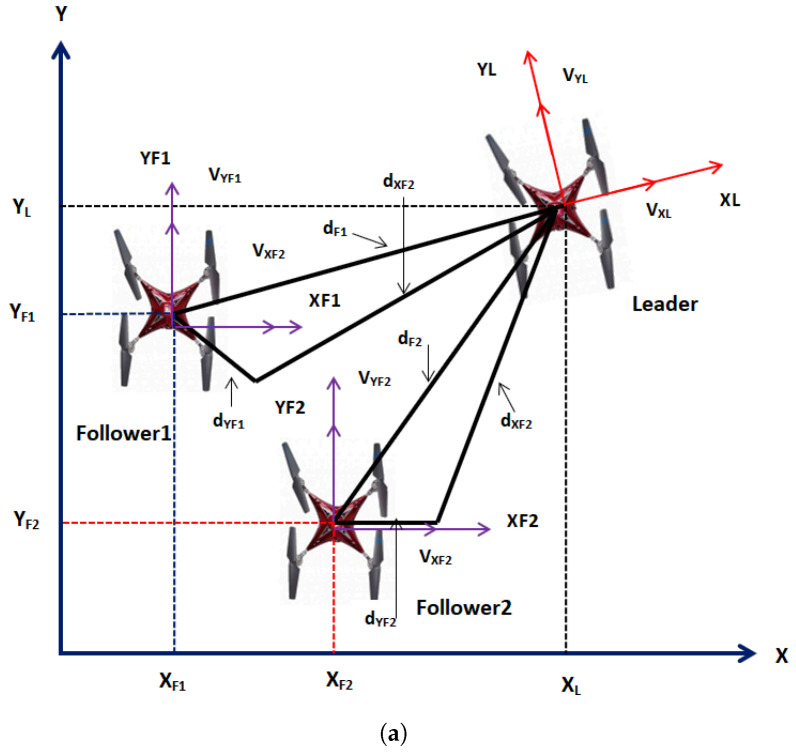

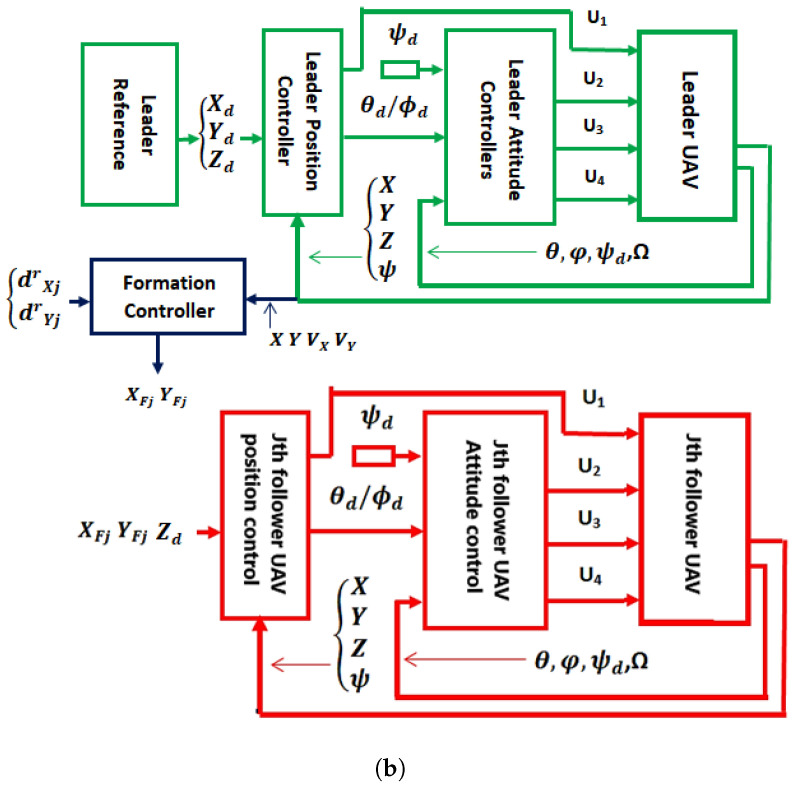
(**a**) Leader-Follower Configuration (**b**) Block diagram.

Figure 2a shows the vector diagram of multiple UAV quad-rotors in leader follower configuration. Based on the above assumptions, the dynamic model of the multiple UAV quad-rotors is formulated as six degrees of freedom equations. The dynamic equations expressing the linear and angular dynamics of the quad-rotors are given as follows:

Equations (1)–(6) formulate the mathematical model of multiple quad-rotors UAV. From Equations (1)–(6), $D_{Xi}$ and $D_{Yi}$ represent the uncertainty in *X* and *Y* acceleration channels, while *i* is an index representing [L,j] and $j=[F_{1},\;\;F_{2}]$. The subscript *L* represents the leader UAV, while $F_{1}$ and $F_{2}$ show follower1 and 2 UAVs respectively.

Referring to Figure 2, the transnational dynamics of the UAVs are expressed as follows:

(7) $${\dot{X}}_{i}=V_{Xi}\cos\left(\psi_{i}\right)-V_{Yi}\sin\left(\psi_{i}\right)$$

(8) $${\dot{Y}}_{i}=V_{Xi}\sin\left(\psi_{i}\right)+V_{Yi}\cos\left(\psi_{i}\right)$$

(9) $${\dot{\psi }}_{L}=\omega_{L}$$

where $V_{Xi}$ and $V_{Yi}$ represents the velocities in *X* and *Y* directions of the inertial frame. As shown in Figure 2, let the follower UAVs maintain $d_{Xj}$ and $d_{Yj}$ distances in *X* and *Y* planes respectively with respect to the leader UAV, so $d_{Xj}$ and $d_{Yj}$ are expressed as follows:

(10) $$d_{Xj}=-\left(X_{L}-X_{j}\right)\cos\left(\psi_{L}\right)-\left(Y_{L}-Y_{j}\right)\sin\left(\psi_{L}\right)$$

(11) $$d_{Yj}=\left(X_{L}-X_{j}\right)\sin\left(\psi_{L}\right)-\left(Y_{L}-Y_{j}\right)\cos\left(\psi_{L}\right)$$

where $d_{Xj}=d_{i}\cos\left(\phi \right)$, $d_{Yj}=d_{i}\sin\left(\phi \right)$ and $X_{j}=\left[X_{F1},\;\;\;X_{F2}\right]$. The error in ψ dynamics as defined as follows: $e_{\psi }=\psi_{j}-\psi_{L}$. By taking the first derivatives of Equations (10) and (11) with respect to time and combining the resultant expressions with Equations (7) and (8) yields the following expressions:

(12) $${\dot{d}}_{Xj}=d_{Yj}\omega_{L}+V_{Xj}\cos\left(e_{\psi }\right)-V_{Yj}\sin\left(e_{\psi }\right)-V_{XL}$$

(13) $${\dot{d}}_{Yj}=-d_{Xj}\omega_{L}+V_{Xj}\sin e_{\psi }+V_{Yj}\cos e_{\psi }-V_{YL}$$

where $V_{Xj}$, $V_{Yj}$, $V_{XL}$ and $V_{YL}$ represent the longitudinal and lateral velocities of the follower1, follower2 and leader UAVs respectively. By defining errors in the longitudinal and lateral dynamics of Equations (12) and (13), the error state equation is represented as follows:

(14) $$\dot{\chi }=F\left(\chi \right)+G\left(\chi \right)v$$

Equation (14) are explained as follows:

(15) $$\chi =\left[\begin{array}{c} e_{Xj}\\ e_{Yj}\\ e_{\psi } \end{array}\right];\dot{\chi }=\left[\begin{array}{c} {\dot{e}}_{Xj}\\ {\dot{e}}_{Yj}\\ {\dot{e}}_{\psi } \end{array}\right];v=\left[\begin{array}{c} V_{Xj}\\ V_{Yj}\\ \omega_{F} \end{array}\right]$$

Also the terms G(χ) and F(χ) are expressed as follows:

(16) $$F\left(\chi \right)=\left[\begin{array}{c} e_{Yj}\omega_{L}+V_{XL}-\omega_{L}{d_{Yj}}^{d}\\ -e_{Xj}\omega_{L}+V_{YL}+\omega_{L}{d_{Xj}}^{d}\\ e_{\psi } \end{array}\right]$$

(17) $$G\left(\chi \right)=\left[\begin{array}{ccc} -ce_{\psi } & se_{\psi } & 0\\ -se_{\psi } & -ce_{\psi } & 0\\ 0 & 0 & 1 \end{array}\right]$$

In Equation (17), $e_{\psi }$ is already defined, while *c* represents cos while *s* is a sin function. Also from Equation (15), we define: $e_{Xj}={d_{Xj}}^{d}-d_{Xj}$ and $e_{Yj}={d_{Yj}}^{d}-d_{Yj}$. Where ${d_{Xj}}^{d}$ and ${d_{Yj}}^{d}$ represent the desired commands. Finally, the desired reference trajectories for follower UAVs are expressed as follows:

(18) $$\begin{array}{c} X_{dj}=X_{L}-d_{Xj}\cos\left(\psi_{L}\right)-d_{Yj}\sin\left(\psi_{L}\right)\\ Y_{dj}=Y_{L}+d_{Xj}\sin\left(\psi_{L}\right)+d_{Yj}\cos\left(\psi_{L}\right) \end{array}$$

## 3. Trajectory and Formation Controllers Formulation

In this section, as a first step, the derivations of the attitude, altitude and position controllers are formulated for the leader UAV. As a second step, the formation controller is derived and based on it, new references are calculated for follower1 and follower2 UAVs. In the last step, the trajectory and attitude controllers of the leader UAV are generalized for follower UAVs. Before deriving the control schemes, the following assumptions are made:

**Assumption** **4.** *It is assumed that the following condition is true for the uncertainty terms:* *$\left|\right|D_{Xi}\left|\right|\leq \Delta_{1i}$; $\left|\right|D_{Yi}\left|\right|\leq \Delta_{2i}$;$\left|\right|D_{Zi}\left|\right|\leq \Delta_{3i}$;$\left|\right|D_{\phi_{i}}\left|\right|\leq \Delta_{4i}$;$\left|\right|D_{\theta_{i}}\left|\right|\leq \Delta_{5i}$;$\left|\right|D_{\psi_{i}}\left|\right|\leq \Delta_{6i}$* *where $\Delta_{1i}$, $\Delta_{2i}$,$\Delta_{3i}$,$\Delta_{4i}$,$\Delta_{5i}$,$\Delta_{6i}$ represent the upper bound of the mentioned uncertainties.*

### 3.1. Leader UAV Control Formulation

In this subsection, the attitude, altitude and position controllers are derived for leader UAV using adaptive super twisting sliding mode control method.

#### 3.1.1. Attitude Control

Attitude controllers regulate the roll, yaw and pitch angles of the UAV. Let the reference Euler angle commands for the leader UAV are set as $\phi_{dL},\theta_{dL,}\psi_{dL}$, then the desired sliding manifold is chosen as follows:

(19) $$S_{\phi_{L}}=k_{1}e_{\phi_{L}}+k_{2}{\dot{e}}_{\phi_{L}}$$

where $S_{\phi_{L}}$ represents the sliding surface for $\phi_{L}$ loop, $k_{1},k_{2}$ are the design constants and the $\phi_{L}$ loop error dynamics are expressed as follows, i.e., $e_{\phi_{L}}=\phi_{L}-\phi_{dL},\;\;{\dot{e}}_{\phi_{L}}=\dot{\phi_{L}}-{\dot{\phi }}_{dL}$. By taking the first time derivative of Equation (19), the following expression is obtained:

(20) $$\dot{S_{\phi_{L}}}=k_{1}\dot{e_{\phi_{L}}}+k_{2}\ddot{e_{\phi_{L}}}$$

Equations (4) and (20) are combined and expressed as follows:

(21) $$\dot{S_{\phi_{L}}}=k_{1}\dot{e_{\phi_{L}}}+k_{2}\left[a_{1L}\dot{\theta_{L}}\dot{\psi_{L}}-a_{2L}\dot{\theta_{L}}\Omega_{rL}+b_{1L}U_{2L}-D_{\phi_{L}}-{\ddot{\phi }}_{dL}\right]$$

In Equation (21), the coefficients are defined as follows, i.e., $a_{1L}=\frac{I_{yL}-I_{zL}}{I_{xL}},\;\;a_{2L}=\frac{J_{rL}}{I_{xL}},\;\;b_{1L}=\frac{l}{I_{xL}}$; then the equivalent control law for $\phi_{L}$ loop is derived as follows:

(22) $$U_{2Leq}=\frac{1}{b_{1L}}\left(\frac{-k_{1}}{k_{2}}\dot{e_{\phi_{L}}}-a_{1L}\dot{\theta_{L}}\dot{\psi_{L}}+a_{2L}\dot{\theta_{L}}\Omega_{rL}+{\ddot{\phi }}_{dL}\right)$$

Using super twisting algorithm, the switching control law is derived as follows:

(23) $$U_{2Lsw}=\frac{-k_{d1}}{b_{1L}}{\left|S_{\phi_{L}}\right|}^{0.5}sgn\left(S_{\phi_{L}}\right)-\frac{k_{d2}}{b_{1L}}\int sgn\left(S_{\phi_{L}}\right)$$

Referring to Equation (22) and (23), the total control action is the sum of equivalent and switching control parts, i.e., $U_{2L}=U_{2Leq}+U_{2Lsw}$. Similar procedure is adopted to derive the pitch and yaw controllers. The sliding surfaces for $\theta_{L}$ and $\psi_{L}$ loops are defined as follows i.e., $S_{\theta_{L}}=k_{3}e_{\theta_{L}}+k_{4}{\dot{e}}_{\theta_{L}}$ and $S_{\psi_{L}}=k_{5}e_{\psi_{L}}+k_{6}{\dot{e}}_{\psi_{L}}$, then the $\theta_{L}$ and $\psi_{L}$ loops controllers are formulated as follows:

(24) $$U_{3Leq}=\frac{1}{b_{2L}}\left(\frac{-k_{3}}{k_{4}}\dot{e_{\theta_{L}}}-a_{3L}\dot{\phi_{L}}\dot{\psi_{L}}+a_{4L}\dot{\phi_{L}}\Omega_{rL}+{\ddot{\theta }}_{dL}\right)$$

(25) $$U_{3Lsw}=\frac{-k_{d3}}{b_{2L}}{\left|S_{\theta_{L}}\right|}^{0.5}sgn\left(S_{\theta_{L}}\right)-\frac{k_{d4}}{b_{2L}}\int sgn\left(S_{\theta_{L}}\right)$$

(26) $$U_{4Leq}=\frac{1}{b_{3L}}\left(\frac{-k_{5}}{k_{6}}\dot{e_{\psi_{L}}}-a_{5L}\dot{\phi_{L}}\dot{\theta_{L}}+{\ddot{\psi }}_{dL}\right)$$

(27) $$U_{4Lsw}=\frac{-k_{d5}}{b_{3L}}{\left|S_{\psi_{L}}\right|}^{0.5}sgn\left(S_{\psi_{L}}\right)-\frac{k_{d6}}{b_{3L}}\int sgn\left(S_{\psi_{L}}\right)$$

From Equations (19)–(27), constant parameters $k_{1},k_{2},k_{3},k_{4},k_{5},k_{6},k_{d1},k_{d2},k_{d3},k_{d4},k_{d5},k_{d6}$ represent controllers and sliding surface gains. The coefficients are defined as follows: $a_{3L}=\frac{I_{zL}-I_{xL}}{I_{yL}},\;\;a_{4L}=\frac{J_{rL}}{I_{yL}},\;\;b_{2L}=\frac{lL}{I_{yL}},$$a_{5L}=\frac{I_{xL}-I_{yL}}{I_{zL}}$ and $\;b_{3L}=\frac{lL}{I_{xL}}$, Moreover, $S_{\theta_{L}}$ and $S_{\psi_{L}}$ represent the sliding surfaces for $\theta_{L}$ and $\psi_{L}$ loops. The corresponding error dynamics for $\theta_{L}$ and $\psi_{L}$ loops are expressed as follows, i.e., $e_{\theta_{L}}=\theta_{L}-\theta_{dL},\;\;{\dot{e}}_{\theta_{L}}=\dot{\theta_{L}}-{\dot{\theta }}_{dL}$, $e_{\psi_{L}}=\psi_{L}-\psi_{dL},\;\;{\dot{e}}_{\psi_{L}}=\dot{\psi_{L}}-{\dot{\psi }}_{dL}$.

**Theorem** **1.** *Consider the nonlinear system presented in Equations (4)–(6),satisfying assumptions 1–3, then under the proposed controllers of Equations (22)–(25), states of the attitude dynamics will converge to the origin in finite time [45].*

**Proof.**  Proof of Theorem 1 [45] The stability proof is only derived for $\phi_{L}$ loop only. Similar procedures can be be adopted for the other two loops of attitude dynamics. Equation (23) is modified as follows: $U_{2Lsw}=\frac{-k_{d1}}{b_{1L}}{\left|S_{\phi_{L}}\right|}^{0.5}sgn\left(S_{\phi_{L}}\right)+v_{\phi L}$; where the term $v_{\phi L}$ is calculated from the following expression: ${\dot{v}}_{\phi L}=\frac{k_{d2}}{b_{1L}}sgn\left(S_{\phi_{L}}\right)$. By combining the above terms with Equations (21) and (22), ${\dot{S}}_{\phi L}$ is expressed as follows:

(28) $$\begin{array}{l} \dot{S_{\phi_{L}}}=\frac{-k_{d1}}{b_{1L}}{\left|S_{\phi_{L}}\right|}^{0.5}sgn\left(S_{\phi_{L}}\right)+v_{\phi L}-D_{\phi L}\\ {\dot{v}}_{\phi L}=-\frac{k_{d2}}{b_{1L}}sgn\left(S_{\phi_{L}}\right) \end{array}$$

Let the Lyapunov function for ϕ loop dynamics is chosen as follows: $V_{\phi_{L}}=2\tau_{2}|S_{\phi_{L}}|+0.5v_{\phi L}^{2}+0.5(\tau_{1}|S_{\phi_{L}}{{|}^{0.5}sgn\left(S_{\phi_{L}}\right)-v_{\phi L})}^{2}$. Where $\tau_{1}=\frac{k_{d1}}{b_{1L}}$ and $\tau_{2}=\frac{k_{d2}}{b_{1L}}$. A new state vector is defined as follows: $\eta_{\phi L}^{T}=\left[\right|S_{\phi_{L}}{|}^{0.5}sgn\left(S_{\phi_{L}}\right)\;\;\;v_{\phi L}]$. Define matrix $P_{\phi L}=\left[\begin{array}{cc} 4\tau_{2}+\tau^{2} & -\tau_{1}\\ -\tau_{1} & 2 \end{array}\right]$ and then the Lyapunov function is expressed as follows: $V_{\phi_{L}}=\eta_{\phi L}^{T}P\eta_{\phi L}$ The time derivative of the Lyapunov function along (28) yields the following relation [45]:

(29) $$\dot{V_{\phi_{L}}}=-\frac{1}{\left|S_{\phi_{L}}^{0.5}\right|}\eta_{\phi L}^{T}Q\eta_{\phi L}+\Delta_{4L}q_{\phi L}^{T}\eta_{\phi L}$$

where the new matrices are represented as follows: $Q_{\phi L}=\frac{\tau_{1}}{2}\left(\begin{array}{cc} 2\tau_{2}+\tau_{1}^{2} & -\tau_{1}\\ -\tau_{1} & 1 \end{array}\right)$ and $q_{\phi L}^{T}=\left(2\tau_{2}+\frac{1}{2}\tau_{1}^{2}-\frac{1}{2}\tau_{1}\right)$. Applying the uncertainty bounds mentioned in Assumption 4, expression (29) is simplified as follows [32]:

(30) $$\dot{V_{\phi L}}=-\frac{\tau_{1}}{2\left|S_{\phi L}^{0.5}\right|}\eta_{\phi L}^{T}\tilde{Q_{\phi L}}\eta_{\phi L}$$

where matrix $\tilde{Q_{\phi L}}=\left(\begin{array}{cc} 2\tau_{2}+\tau_{1}^{2}-\left(\frac{4\tau_{2}}{\tau_{1}}+\tau_{1}\right)\Delta_{4L} & -\tau_{1}+2\Delta_{4L}\\ -\tau_{1}+2\Delta_{4L} & 1 \end{array}\right)$. Equation (30) is negative definite only if $\tilde{Q_{\phi L}}>0$. If the gains satisfy the following criteria $\tau_{1}>2\Delta_{4L},\;\tau_{2}>\tau_{1}\frac{5\Delta_{4L}\tau_{1}+4\Delta_{4L}^{2}}{2\left(\tau_{1}-2\Delta_{4L}\right)}$, then $\tilde{Q}>0$ and ${\dot{V}}_{\phi_{L}}<0$.

**Remark** **1.** *The proof of finite time convergence property can be derived by using the procedures adopted in [45].* □

#### 3.1.2. Altitude and Position Control

This section formulates the altitude and position control system for the leader UAV expressed in Equations (1)–(6). First the altitude control system is derived and then using the transformation matrix, the position controllers are formulated. With the desired altitude $Z_{dL}$, the sliding manifold is written as follows:

(31) $$S_{Z_{L}}=k_{7}e_{Z_{L}}+k_{8}{\dot{e}}_{Z_{L}}$$

In Equation (31), $k_{7}$ and $k_{8}$ represent the design constant. The error dynamics are defined as follows: i.e., $e_{Z_{L}}=Z_{L}-Z_{d_{L}},\;{\dot{e}}_{Z_{L}}=\dot{Z_{L}}-{\dot{Z_{L}}}_{d_{L}}$. Taking the first time derivative of Equation (32) yields the following expression:

(32) $$\dot{S_{Z_{L}}}=k_{7}\dot{e_{Z_{L}}}+k_{8}{\ddot{e}}_{Z_{L}}$$

Equation (32) and Equation (3) are combined and expressed as follows:

(33) $$\dot{S_{Z_{L}}}=k_{7}\dot{e_{Z_{L}}}+k_{8}\left[g-\cos\theta_{L}\cos\phi_{L}\frac{U_{1L}}{m_{QL}}-D_{ZL}-{\ddot{Z}}_{dL}\right]$$

Using super twisting sliding mode theory, the altitude controller is derived as follows:

(34) $$\begin{array}{c} U_{Z_{L}}=-\frac{k_{7}}{k_{8}}\dot{e_{Z_{L}}}+\ddot{Z_{dL}}-\frac{k_{d7}}{k_{8}}{\left|S_{Z_{L}}\right|}^{0.5}sgn\left(S_{Z_{L}}\right)-\frac{k_{d8}}{k_{8}}\int sgn\left(S_{Z_{L}}\right)\\ U_{1L}=\frac{m_{QL}}{\cos\theta_{L}\cos\phi_{L}}[g-(-\frac{k_{7}}{k_{8}}\dot{e_{Z_{L}}}+\ddot{Z_{dL}}-\frac{k_{d7}}{k_{8}}|S_{Z_{L}}{|}^{0.5}sgn\left(S_{Z_{L}}\right)-\frac{k_{d8}}{k_{8}}\int sgn\left(S_{Z_{L}}\right))] \end{array}$$

Here $U_{Z_{L}}=\ddot{Z_{L}}=g-\left(\cos\theta_{L}\cos\phi_{L}\right)\frac{U_{1L}}{m_{QL}}$ represents the virtual control law. The stability proof is derived based on the same concepts presented for ϕ loop. The robust terms of Equation (34) are modified as follows: $U_{1Lsw}=\frac{-k_{d7}}{k_{8}}{\left|S_{Z_{L}}\right|}^{0.5}sgn\left(S_{Z_{L}}\right)+v_{Z_{L}}$; where the term $v_{Z_{L}}$ is calculated from the following expression: ${\dot{v}}_{Z_{L}}=-\frac{k_{d8}}{k_{8}}sgn\left(S_{Z_{L}}\right)$. By combining the above terms with Equation (33) and robust term of (34), ${\dot{S}}_{ZL}$ is expressed as follows:

(35) $$\begin{array}{l} \dot{S_{Z_{L}}}=\frac{-k_{d7}}{k_{8}}{\left|S_{Z_{L}}\right|}^{0.5}sgn\left(S_{Z_{L}}\right)+v_{Z_{L}}-D_{ZL}\\ {\dot{v}}_{Z_{L}}=-\frac{k_{d8}}{k_{8}}sgn\left(S_{Z_{L}}\right) \end{array}$$

Let the Lyapunov function for *Z* loop is chosen as follows: $V_{Z_{L}}=2\tau_{8}|S_{Z_{L}}|+0.5v_{Z_{L}}^{2}+0.5(\tau_{7}|S_{Z_{L}}{{|}^{0.5}sgn\left(S_{Z_{L}}\right)-v_{Z_{L}})}^{2}$. Where $\tau_{7}=\frac{k_{d7}}{k_{8}}$ and $\tau_{8}=\frac{k_{d8}}{k_{8}}$. A new state vector is defined as follows: $\eta_{ZL}^{T}=\left[\right|S_{Z_{L}}{|}^{0.5}sgn\left(S_{Z_{L}}\right)\;\;\;v_{Z_{L}}]$. Define matrix $P_{ZL}=\left[\begin{array}{cc} 4\tau_{8}+\tau_{7}^{2} & -\tau_{7}\\ -\tau_{7} & 2 \end{array}\right]$ and then the Lyapunov function is expressed as follows: $V_{Z_{L}}=\eta_{ZL}^{T}P_{ZL}\eta_{ZL}$ The time derivative of the Lyapunov function along (35) yields the following relation [45]:

(36) $$\dot{V_{Z_{L}}}=-\frac{1}{\left|S_{Z_{L}}^{0.5}\right|}\eta_{ZL}^{T}Q_{ZL}\eta_{ZL}+\Delta_{3L}q_{ZL}^{T}\eta_{ZL}$$

where the new matrices are represented as follows: $Q_{ZL}=\frac{\tau_{7}}{2}\left(\begin{array}{cc} 2\tau_{8}+\tau_{7}^{2} & -\tau_{7}\\ -\tau_{7} & 1 \end{array}\right)$ and $q_{ZL}^{T}=\left(2\tau_{8}+\frac{1}{2}\tau_{7}^{2}-\frac{1}{2}\tau_{7}\right)$. Applying the uncertainty bounds given in Assumption 4, expression (36) is simplified as follows [45]:

(37) $$\dot{V_{ZL}}=-\frac{\tau_{7}}{2\left|S_{ZL}^{0.5}\right|}\eta_{ZL}^{T}\tilde{Q_{ZL}}\eta_{ZL}$$

where matrix $\tilde{Q_{ZL}}=\left(\begin{array}{cc} 2\tau_{8}+\tau_{7}^{2}-\left(\frac{4\tau_{8}}{\tau_{7}}+\tau_{7}\right)\Delta_{3L} & -\tau_{7}+2\Delta_{3L}\\ -\tau_{7}+2\Delta_{3L} & 1 \end{array}\right)$. Equation (37) is negative definite only if $\tilde{Q_{ZL}}>0$. If the gains satisfy the following criteria $\tau_{7}>2\Delta_{3L},\;\tau_{8}>\tau_{7}\frac{5\Delta_{3L}\tau_{7}+4\Delta_{3L}^{2}}{2\left(\tau_{7}-2\Delta_{3L}\right)}$, then $\tilde{Q_{ZL}}>0$ and ${\dot{V}}_{Z_{L}}<0$.

Now to derive the XY controllers, we assume the following:

$U_{XL}=\left(\sin\psi_{L}\sin\phi_{L}+\cos\psi_{L}\sin\theta_{L}\cos\phi_{L}\right)\frac{U_{1L}}{m_{QL}}$

$U_{YL}=\left(-\cos\psi_{L}\sin\phi_{L}+\sin\psi_{L}\sin\theta_{L}\cos\phi_{L}\right)\frac{U_{1L}}{m_{QL}}$.

With these expressions, Equations (1) and (2) are re-written for leader UAV in the following form:

(38) $$\ddot{X_{L}}=U_{XL}-D_{XL}$$

(39) $$\ddot{Y_{L}}=U_{YL}-D_{YL}$$

Let the sliding manifolds for the position loops of leader UAV are expressed as follows:

(40) $$\left\{\begin{array}{c} S_{X_{L}}=k_{9}e_{X_{L}}+k_{10}{\dot{e}}_{X_{L}}\\ S_{Y_{L}}=k_{11}e_{Y_{L}}+k_{12}{\dot{e}}_{Y_{L}} \end{array}\right.$$

In Equation (40) $k_{9}$,$k_{10}$,$k_{11}$,$k_{12}$ are the design constants and the error dynamics are expressed as follows: $e_{X_{L}}=X_{L}-X_{dL},\;\;{\dot{e}}_{X_{L}}=\dot{X_{L}}-{\dot{X}}_{dL},\;e_{Y_{L}}=Y_{L}-Y_{dL},\;\;{\dot{e}}_{Y_{L}}=\dot{Y_{L}}-{\dot{Y}}_{dL}$. By taking the first time derivative of Equation 40, and combining it with Equations (38) and (39) one acquires the following expressions:

(41) $$\left\{\begin{array}{c} \dot{S_{X_{L}}}=k_{9}\dot{e_{X_{L}}}+k_{10}\left[U_{XL}-D_{XL}-{\ddot{X}}_{dL}\right]\\ \dot{S_{Y_{L}}}=k_{11}\dot{e_{Y_{L}}}+k_{12}\left[U_{YL}-D_{YL}-{\ddot{Y}}_{dL}\right] \end{array}\right.$$

From Equation (41), the virtual controllers $U_{XL}$ and $U_{YL}$ are expressed as follows:

(42) $$\begin{array}{c} U_{XL}=({\ddot{X}}_{dL}-\frac{k_{9}}{k_{10}}\dot{e_{X_{L}}}-\frac{k_{d9}}{k_{10}}|S_{X_{L}}{|}^{0.5}sgn\left(S_{X_{L}}\right)-\frac{k_{d10}}{k_{10}}\int sgn\left(S_{X_{L}}\right))\\ U_{YL}=({\ddot{Y}}_{dL}-\frac{k_{11}}{k_{12}}\dot{e_{Y_{L}}}-\frac{k_{d11}}{k_{12}}|S_{Y_{L}}{|}^{0.5}sgn\left(S_{Y_{L}}\right)-\frac{k_{d12}}{k_{12}}\int sgn\left(S_{Y_{L}}\right)) \end{array}$$

The stability proof is derived based on the same concepts presented for *Z* loop. The robust terms of Equation (42) are modified as follows: $U_{XLsw}=\frac{-k_{d9}}{k_{10}}{\left|S_{X_{L}}\right|}^{0.5}sgn\left(S_{X_{L}}\right)+v_{X_{L}}$; where the term $v_{X_{L}}$ is calculated from the following expression: ${\dot{v}}_{X_{L}}=-\frac{k_{d10}}{k_{10}}sgn\left(S_{X_{L}}\right)$ and $U_{YLsw}=\frac{-k_{d11}}{k_{12}}{\left|S_{Y_{L}}\right|}^{0.5}sgn\left(S_{Y_{L}}\right)+v_{Y_{L}}$; where the term $v_{Y_{L}}$ is calculated from the following expression: ${\dot{v}}_{Y_{L}}=-\frac{k_{d11}}{k_{12}}sgn\left(S_{Y_{L}}\right)$. By combining the above terms with Equations (41) and (42), ${\dot{S}}_{XL}$ and ${\dot{S}}_{YL}$ are expressed as follows:

(43) $$\begin{array}{c} \;\;\;\;\;\;\;\;\;\;\;\;\;\;\;\;\;\;\;\;\;\;\;\;\;\;\;\;\;\;\;\;\;\;\;\;\;\;\;\;\;\;\;\;\;\;\;\;\;\;\;\;\;\;\;\;\;\;\;\;\;\;\;\;\;\;\;\;\;\;\;\;\;\;\;\;\;\;\dot{S_{X_{L}}}=\frac{-k_{d9}}{k_{10}}{\left|S_{X_{L}}\right|}^{0.5}sgn\left(S_{X_{L}}\right)+v_{X_{L}}-D_{XL}\\ {\dot{v}}_{X_{L}}=-\frac{k_{d10}}{k_{10}}sgn\left(S_{X_{L}}\right)\\ \;\;\;\;\;\;\;\;\;\;\;\;\;\;\;\;\;\;\;\;\;\;\;\;\;\;\;\;\;\;\;\;\;\;\;\;\;\;\;\;\;\;\;\;\;\;\;\;\;\;\;\;\;\dot{S_{YLL}}=\frac{-k_{d11}}{k_{12}}{\left|S_{Y_{L}}\right|}^{0.5}sgn\left(S_{Y_{L}}\right)+v_{Y_{L}}-D_{YL}\\ {\dot{v}}_{Y_{L}}=-\frac{k_{d12}}{k_{12}}sgn\left(S_{Y_{L}}\right)\;\;\;\;\;\;\;\;\;\;\;\;\;\;\;\;\;\;\;\;\;\;\;\;\;\;\;\;\;\;\;\;\;\;\;\;\;\;\;\;\;\;\;\;\;\;\;\;\;\;\;\;\;\;\;\;\;\;\;\;\;\;\;\;\;\;\; \end{array}$$

Let the Lyapunov function for *X* loop dynamics is chosen as follows: $V_{X_{L}}=2\tau_{10}|S_{X_{L}}|+0.5v_{X_{L}}^{2}+0.5(\tau_{9}|S_{X_{L}}{{|}^{0.5}sgn\left(S_{X_{L}}\right)-v_{X_{L}})}^{2}$, where as for *Y* loop dynamics is the Lyapunov function is given as follows: $V_{Y_{L}}=2\tau_{12}|S_{Y_{L}}|+0.5v_{Y_{L}}^{2}+0.5(\tau_{11}|S_{Y_{L}}{{|}^{0.5}sgn\left(S_{Y_{L}}\right)-v_{Y_{L}})}^{2}$. Where $\tau_{9}=\frac{k_{d9}}{k_{10}}$, $\tau_{10}=\frac{k_{d10}}{k_{10}}$,$\tau_{11}=\frac{k_{d11}}{k_{12}}$, $\tau_{12}=\frac{k_{d12}}{k_{12}}$. The following new state vectors are defined: $\eta_{XL}^{T}=\left[\right|S_{X_{L}}{|}^{0.5}sgn\left(S_{X_{L}}\right)\;\;\;v_{X_{L}}]$; $\eta_{YL}^{T}=\left[\right|S_{Y_{L}}{|}^{0.5}sgn\left(S_{Y_{L}}\right)\;\;\;v_{Y_{L}}]$. Define new matrices as follows: $P_{XL}=\left[\begin{array}{cc} 4\tau_{10}+\tau_{9}^{2} & -\tau_{9}\\ -\tau_{9} & 2 \end{array}\right]$; $P_{YL}=\left[\begin{array}{cc} 4\tau_{12}+\tau_{11}^{2} & -\tau_{11}\\ -\tau_{11} & 2 \end{array}\right]$ and then the Lyapunov functions are expressed as follows: $V_{X_{L}}=\zeta_{1}\eta_{XL}^{T}P_{XL}\eta_{XL}+D_{XL}^{T}D_{XL}$; $V_{Y_{L}}=\zeta_{2}\eta_{YL}^{T}P_{YL}\eta_{YL}+D_{YL}^{T}D_{YL}$ The time derivative of the Lyapunov functions along (43) yields the following relation [45]:

(44) $$\left[\begin{array}{c} \dot{V_{X_{L}}}=-\zeta_{1}\frac{1}{\left|S_{X_{L}}^{0.5}\right|}\eta_{XL}^{T}Q_{XL}\eta_{XL}+\zeta_{1}D_{XL}q_{XL}^{T}\eta_{XL}+D_{XL}^{T}{\dot{D}}_{XL}\\ \dot{V_{Y_{L}}}=-\zeta_{2}\frac{1}{\left|S_{Y_{L}}^{0.5}\right|}\eta_{YL}^{T}Q_{YL}\eta_{YL}+\zeta_{2}D_{YL}q_{YL}^{T}\eta_{YL}+D_{YL}^{T}{\dot{D}}_{YL} \end{array}\right]$$

where: $Q_{XL}=\frac{\tau_{9}}{2}\left(\begin{array}{cc} 2\tau_{10}+\tau_{9}^{2} & -\tau_{9}\\ -\tau_{9} & 1 \end{array}\right)$; $Q_{YL}=\frac{\tau_{11}}{2}\left(\begin{array}{cc} 2\tau_{12}+\tau_{11}^{2} & -\tau_{11}\\ -\tau_{11} & 1 \end{array}\right)$ and $q_{XL}^{T}=\left(2\tau_{10}+\frac{1}{2}\tau_{9}^{2}\;\;-\frac{1}{2}\tau_{9}\right)$;$q_{YL}^{T}=\left(2\tau_{12}+\frac{1}{2}\tau_{11}^{2}\;\;-\frac{1}{2}\tau_{11}\right)$. From Equation (44), since $D_{XL}$ and $D_{YL}$ are scalar quantities so $D_{XL}^{T}=D_{XL}$ and $D_{YL}^{T}=D_{YL}$, then adaptive laws are derived as follows:

(45) $$\begin{array}{cc} \; & \dot{D_{XL}}=-\zeta_{1}q_{XL}^{T}\eta_{XL}\\ \; & \dot{D_{YL}}=-\zeta_{2}q_{YL}^{T}\eta_{YL} \end{array}$$

Applying the uncertainty bounds given in Assumption 4, and by combining Equation (44) with Equation (45), the simplified expressions of (44) are given as follows [45]:

(46) $$\left[\begin{array}{c} \dot{V_{XL}}=-\frac{\tau_{9}}{2\left|S_{XL}^{0.5}\right|}\eta_{XL}^{T}{\tilde{Q}}_{XL}\eta_{XL}\\ \dot{V_{YL}}=-\frac{\tau_{11}}{2\left|S_{YL}^{0.5}\right|}\eta_{YL}^{T}{\tilde{Q}}_{YL}\eta_{YL} \end{array}\right]$$

Here the matrices are defined as follows:

$\tilde{Q_{XL}}=\left(\begin{array}{cc} 2\tau_{10}+\tau_{9}^{2}-\left(\frac{4\tau_{10}}{\tau_{9}}+\tau_{9}\right)E\Delta_{1L} & -\tau_{9}+2E\Delta_{1L}\\ -\tau_{9}+2E\Delta_{1L} & 1 \end{array}\right)$

and

$\tilde{Q_{YL}}=\left(\begin{array}{cc} 2\tau_{12}+\tau_{11}^{2}-\left(\frac{4\tau_{12}}{\tau_{11}}+\tau_{11}\right)E\Delta_{2L} & -\tau_{11}+2E\Delta_{2L}\\ -\tau_{11}+2E\Delta_{2L} & 1 \end{array}\right)$.

The expressions $\dot{V_{XL}}$ and $\dot{V_{YL}}$ are negative definite only if $\tilde{Q_{XL}}>0$ and $\tilde{Q_{yL}}>0$. If the gains satisfy the following criteria $\tau_{9}>2E\Delta_{1L},\;\tau_{10}>\tau_{9}\frac{5E\Delta_{1L}\tau_{9}+4E\Delta_{1L}^{2}}{2\left(\tau_{9}-2E\Delta_{1L}\right)}$; $\tau_{11}>2E\Delta_{2L},\;\tau_{12}>\tau_{11}\frac{5E\Delta_{2L}\tau_{11}+4E\Delta_{2L}^{2}}{2\left(\tau_{11}-2E\Delta_{2L}\right)}$, then $\tilde{Q_{XL}}>0$;$\tilde{Q_{YL}}>0$ and ${\dot{V}}_{X_{L}}<0$;${\dot{V}}_{Y_{L}}<0$. Here the terms $E\Delta_{1L}=D_{XL_{estimated}}-D_{XL}$; $E\Delta_{2L}=D_{YL_{estimated}}-D_{YL}$ represent estimation error of the adaptive loops.

**Remark** **2.** *Discontinuous projection operator is used to implement the adaptive laws $\dot{D_{XL}}$,$\dot{D_{YL}}$. The projection operator is defined as follows:*

(47) $$projD_{(X,Y)L}\left(★\right)=\left\{\begin{array}{cc} 0 & if\;\;\;\;D_{(X,Y)L}=D_{{(X,Y)L}_{max}}\;\;\;\;;\;\;\;★>0\\ 0 & if\;\;\;\;D_{(X,Y)L}=D_{{(X,Y)L}_{min}}\;\;\;\;;\;\;\;★<0\\ ★ & otherwise \end{array}\right.$$

In Equation (45), $\zeta_{1}$ and $\zeta_{2}$ represent the adaptation gains. To generate reference trajectories for $\theta_{dL}$ and $\phi_{dL}$, the virtual controllers $U_{XL}$ and $U_{YL}$ are expressed as follows:

(48) $$\frac{U_{XL}m_{QL}}{U_{1L}}=\cos\psi_{L}\sin\theta_{L}\cos\phi_{L}+\sin\phi_{L}\sin\psi_{L}$$

(49) $$\frac{U_{YL}m_{QL}}{U_{1L}}=\sin\psi_{L}\sin\theta_{L}\cos\phi_{L}-\cos\psi_{L}\sin\phi_{L}$$

Multiplying Equation (48) by sinψ and Equation (49) by cosψ and the deference of the resultant equations yields the following expression:

(50) $$\frac{U_{XL}m_{QL}}{U_{1L}}\sin\psi -\frac{U_{YL}m_{QL}}{U_{1L}}\cos\psi =\sin\phi_{dL}$$

Equation (50) is simplified to get the reference command for ϕ loop of the leader UAV as follows:

(51) $$\phi_{dL}=\sin^{-1}\left[\frac{U_{XL}m_{QL}}{U_{1L}}\sin\psi -\frac{U_{YL}m_{QL}}{U_{1L}}\cos\psi \right]$$

Multiplying Equation (48) by cosψ and Equation (49) by sinψ and the summation of the resultant equations yields the following expression:

(52) $$\frac{U_{XL}m_{QL}}{U_{1L}}\cos\psi +\frac{U_{YL}m_{QL}}{U_{1L}}\sin\psi =\sin\theta_{dL}\cos\phi_{L}$$

Squaring Equation (50) on both hand sides and equating $\sin^{2}\phi_{L}=1-\cos^{2}\phi_{L}$, the expression is written in terms of $\cos\phi_{L}$ and given as follows:

(53) $$\cos\phi_{dL}=\sqrt{1-{\left[\frac{U_{XL}m_{QL}}{U_{1L}}\sin\psi -\frac{U_{YL}m_{QL}}{U_{1L}}\cos\psi \right]}^{2}}$$

Now form Equations (51) and (52), reference command for $\theta_{dL}$ is expressed as follows:

(54) $$\theta_{dL}=\sin^{-1}\left[\frac{\frac{U_{XL}m_{QL}}{U_{1L}}\cos\psi +\frac{U_{YL}m_{QL}}{U_{1L}}\sin\psi }{\sqrt{1-{\left[\frac{U_{XL}m_{QL}}{U_{1L}}\sin\psi -\frac{U_{YL}m_{QL}}{U_{1L}}\cos\psi \right]}^{2}}}\right]$$

### 3.2. Leader Followers Formation Control

Before discussing the trajectory controllers for the followers UAV, it is necessary to derive the formation controller which will generate the desired trajectory for the followers UAV. Let the following sliding surface is defined for formation controller:

(55) $$S_{\chi_{j}}=\chi_{j}+\tau \int \chi_{j}$$

where τ represents gain matrix of the sliding surface. By taking time derivative of Equation (55) and combining it with Equation (14), one obtains the following expression:

(56) $$\dot{S_{\chi_{j}}}=F\left(\chi_{j}\right)+G\left(\chi_{j}\right)vj+\tau \chi_{j}$$

For formation control, the desired longitudinal and lateral velocities of the followers UAV are calculated as follows:

(57) $$vj_{eq}=G{\left(\chi_{j}\right)}^{-1}\left[-F\left(\chi_{j}\right)-\tau \chi_{j}\right]$$

(58) $$vj_{sw}=-\eta_{1}{\left|S_{\chi_{j}}\right|}^{0.5}sgn\left(S_{\chi_{j}}\right)-\eta_{2}\int sgn\left(S_{\chi_{j}}\right)$$

For the stability proof, the same procedures as adopted for XYZ loops are applied here.

### 3.3. Followers UAV Control Formulation

In this section, we briefly explain the trajectory control of followers UAV.As mentioned above, the reference position trajectories are generated using Equation (18), and governed by the formation controller of Equations (57) and (58). Thus, by defining the attitude, altitude and position errors for the followers UAV, the rest of the analysis used for the derivation of the subject controllers is the same as leader UAV. For simplicity, here the final control laws are included: let the attitude sliding manifolds are defined as follows:

(59) $$\begin{array}{cc} \; & S_{\phi_{j}}=k_{1j}e_{\phi_{j}}+k_{2j}{\dot{e}}_{\phi_{j}}\\ \; & S_{\theta_{j}}=k_{3j}e_{\theta_{j}}+k_{4j}{\dot{e}}_{\theta_{j}}\\ \; & S_{\psi_{j}}=k_{5j}e_{\psi_{j}}+k_{6j}{\dot{e}}_{\psi_{j}} \end{array}$$

where $j=[F_{1},\;\;F_{2}]$ and $F_{1}$ and $F_{2}$ represent follower1 and follower2 UAVs respectively. Also $k_{1j}$,$k_{2j}$,$k_{3j}$,$k_{4j}$,$k_{5j}$,$k_{6j}$ represent the constants of sliding surfaces for followers UAV. The respective errors are defined as follows: $e_{\phi_{j}}=\phi_{j}-\phi_{dj}$; $e_{\theta_{j}}=\theta_{j}-\theta_{dj}$; $e_{\psi_{j}}=\psi_{j}-\psi_{dj}$. Similarly position and altitude sliding manifolds for follower1 and 2 are given as follows:

(60) $$\begin{array}{cc} \; & S_{Zj}=k_{7j}e_{Zj}+k_{8j}\dot{e_{Zj}}\\ \; & S_{Xj}=k_{9j}e_{Xj}+k_{10j}\dot{e_{Xj}}\\ \; & S_{Yj}=k_{11j}e_{Yj}+k_{12j}\dot{e_{Yj}} \end{array}$$

where $k_{7j}$,$k_{8j}$,$k_{9j}$,$k_{10j}$,$k_{11j}$,$k_{12j}$ represent the constants of sliding surfaces for followers UAV. The respective errors are defined as follows: $e_{Z_{j}}=Z_{j}-Z_{dj}$; $e_{X_{j}}=X_{j}-X_{dj}$; $e_{Y_{j}}=Y_{j}-Y_{dj}$. Now following the same procedures, the attitude, altitude and position controllers for followers UAV are formulated as follows:

(61) $$U_{2jeq}=\frac{1}{b_{1j}}\left(\frac{-k_{1j}}{k_{2j}}\dot{e_{\phi_{j}}}-a_{1j}\dot{\theta_{j}}\dot{\psi_{j}}+a_{2j}\dot{\theta_{j}}\Omega_{rj}+{\ddot{\phi }}_{dj}\right)$$

(62) $$U_{2jsw}=\frac{-k_{d1j}}{b_{1j}}{\left|S_{\phi_{j}}\right|}^{0.5}sgn\left(S_{\phi_{j}}\right)-\frac{k_{d2j}}{b_{1j}}\int sgn\left(S_{\phi_{j}}\right)$$

(63) $$U_{3jeq}=\frac{1}{b_{2j}}\left(\frac{-k_{3j}}{k_{4j}}\dot{e_{\theta_{j}}}-a_{3j}\dot{\phi_{j}}\dot{\psi_{j}}+a_{4j}\dot{\phi_{j}}\Omega_{rj}+{\ddot{\theta }}_{dj}\right)$$

(64) $$U_{3jsw}=\frac{-k_{d3j}}{b_{2j}}{\left|S_{\theta_{j}}\right|}^{0.5}sgn\left(S_{\theta_{j}}\right)-\frac{k_{d4j}}{b_{2j}}\int sgn\left(S_{\theta_{j}}\right)$$

(65) $$U_{4jeq}=\frac{1}{b_{3j}}\left(\frac{-k_{5j}}{k_{6j}}\dot{e_{\psi_{j}}}-a_{5j}\dot{\phi_{j}}\dot{\theta_{j}}+{\ddot{\psi }}_{dj}\right)$$

(66) $$U_{4jsw}=\frac{-k_{d5j}}{b_{3j}}{\left|S_{\psi_{j}}\right|}^{0.5}sgn\left(S_{\psi_{j}}\right)-\frac{k_{d6j}}{b_{3j}}\int sgn\left(S_{\psi_{j}}\right)$$

(67) $$\begin{array}{cc} \; & U_{1j}=\frac{m_{Qj}}{\cos\theta_{j}\cos\phi_{j}}[g-(-\frac{k_{7}j}{k_{8j}}\dot{e_{Z_{j}}}+\ddot{Z_{dj}}\\ & -\frac{k_{d7j}}{k_{8j}}|S_{Z_{j}}{|}^{0.5}sgn\left(S_{Z_{j}}\right)-\frac{k_{d8j}}{k_{8j}}\int sgn\left(S_{Z_{j}}\right))] \end{array}$$

(68) $$\begin{array}{c} U_{Xj}=({\ddot{X}}_{dj}-\frac{k_{9j}}{k_{10j}}\dot{e_{X_{j}}}-\frac{k_{d9j}}{k_{10j}}|S_{X_{j}}{|}^{0.5}sgn\left(S_{X_{j}}\right)-\frac{k_{d10j}}{k_{10j}}\int sgn\left(S_{X_{j}}\right))\\ U_{yj}=({\ddot{Y}}_{dj}-\frac{k_{11j}}{k_{12j}}\dot{e_{Y_{j}}}-\frac{k_{d11j}}{k_{1}2j}|S_{Y_{j}}{|}^{0.5}sgn\left(S_{Y_{j}}\right)-\frac{k_{d12j}}{k_{12j}}\int sgn\left(S_{Y_{j}}\right)) \end{array}$$

(69) $$\begin{array}{cc} \; & \dot{D_{Xj}}=-\zeta_{1}jS_{X_{j}}\\ \; & \dot{D_{Yj}}=-\zeta_{2}jS_{Y_{j}} \end{array}$$

The reference commands for $\theta_{dj}$ and $\phi_{dj}$ are derived using the same procedures given in Equations (48)–(54).

## 4. Results and Discussion

**Figure 3:**
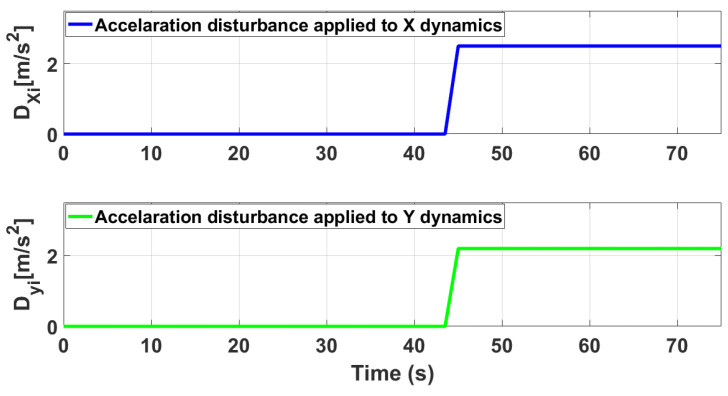
Applied acceleration type disturbance in *X* and *Y* dynamics.

In this section, the proposed ASTSMC controller is tested numerically for the system of multiple quad-rotors shown in Figure 2. The parameters of the leader and followers UAVs are identical and given in Table 1. The control parameters of leader and follower UAVs are given in Table 2 and Table 3. Since the UAVs are identical, leader and followers UAV use the same control parameters.The parameters for formation control loops are chosen as follows: $\tau_{F1}=\tau_{F2}=\left[\begin{array}{c} 1.5\\ 1.5\\ 0.5 \end{array}\right]$, $\eta_{1F1}=\eta_{1F2}=\left[\begin{array}{c} 0.1\\ 0.1\\ 0.075 \end{array}\right]$; $\eta_{2F1}=\eta_{2F2}=\left[\begin{array}{c} 0.05\\ 0.05\\ 0.02 \end{array}\right]$. For the leader UAV, the reference position and altitude commands are set as follows: $X_{L}=\sin t$, $Y_{L}=\cos t$ and $Z_{L}=t$. Figure 3 shows the applied acceleration disturbance on *X* and *Y* dynamics of the leader and followers UAV. it is assumed that same type of disturbance acceleration is applied for all UAVs. Furthermore the disturbance acceleration has no effect on the *Z* dynamics of UAV. Moreover, the following parametric uncertainties are applied: $a_{1L}=2.5a_{1L}$;$a_{1j}=2.5a_{1j}$;$a_{2L}=2.5a_{2L}$;$a_{2j}=2.5a_{2j}$;$a_{3L}=2.5a_{3L}$;$a_{3j}=2.5a_{3j}$;$a_{4L}=2.5a_{4L}$;$a_{4j}=2.5a_{4j}$;$a_{5L}=2.5a_{5L}$;$a_{5j}=2.5a_{5j}$;$b_{1L}=1.75b_{1L}$;$b_{1j}=1.75b_{1j}$;$b_{2L}=1.75b_{2L}$;$b_{2j}=1.75b_{2j}$;$b_{3L}=1.75b_{3L}$;$b_{3j}=1.75b_{3j}$.

**Figure 4:**
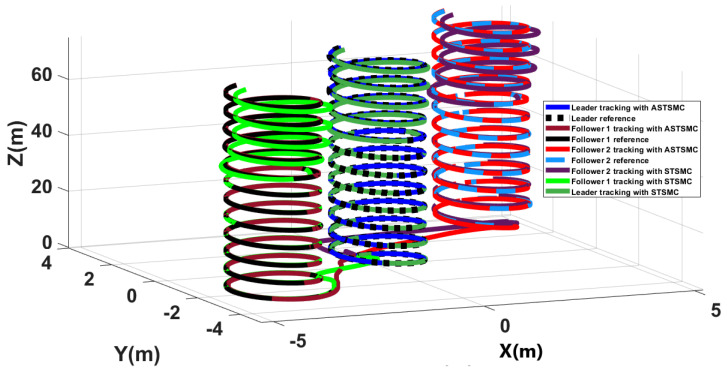
XYZ trajectory tracking comparison under wind disturbance.

Figure 4 shows the trajectory tracking simulations of leader followers UAV in presence of applied disturbance of Figure 3. From the presented results, it is concluded that in presence of disturbances, ASTSMC controllers ensure robust behaviour, while the fixed gain STSMC controllers exhibit steady state errors in the *X* and *Y* tracking responses of the leader and followers UAVs.

**Figure 5:**
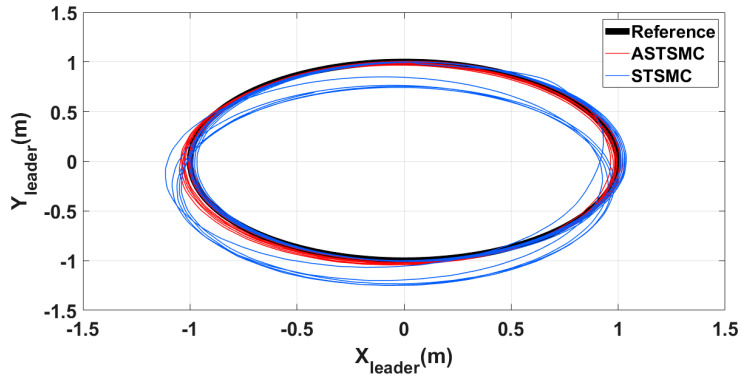
XY leader trajectory tracking comparison under wind disturbance.

**Figure 6:**
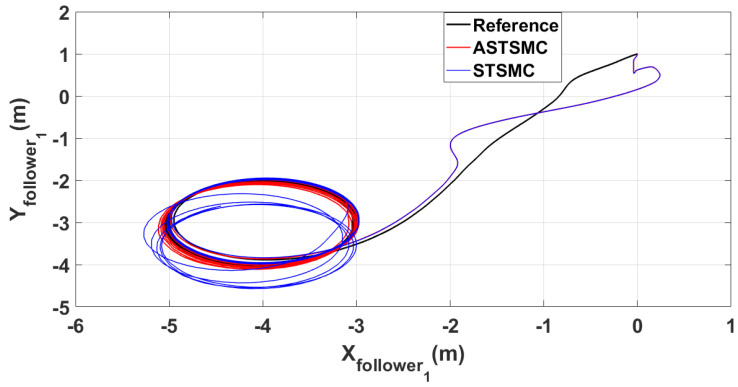
XY follower1 trajectory tracking comparison under wind disturbance.

**Figure 7:**
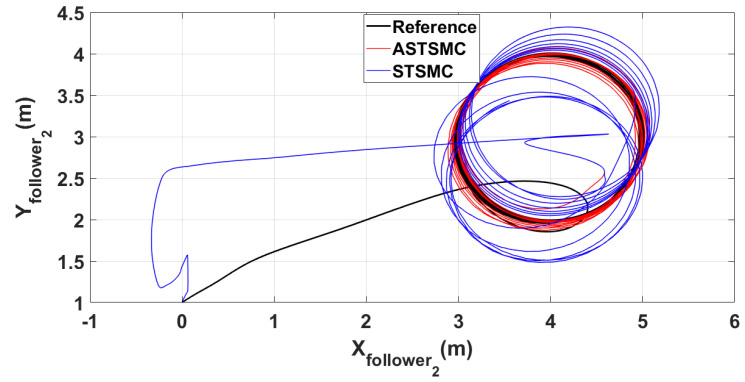
XY follower2 trajectory tracking comparison under wind disturbance.

**Figure 8:**
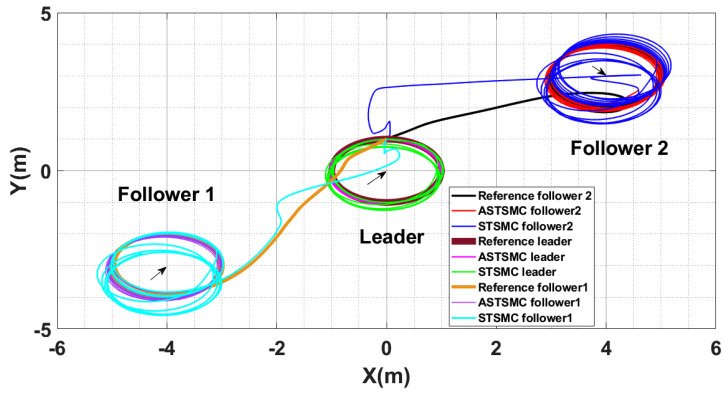
XY Leader-followers trajectory tracking comparison under wind disturbance.

To have a clear picture of the trajectory deviations under wind disturbance, Figure 5, Figure 6, Figure 7 and Figure 8 show the trajectory tracking comparisons in XY plane for leader, follower1 and follower2 UAVs respectively. From the presented results, it is concluded that minimum deviations are observed in the trajectory tracking for all UAVs with ASTSMC controllers, while with fixed gain STSMC controllers all UAVs show significant drift from the reference trajectories in XY plane. Figure 8 shows the combined trajectories of leader followers UAVs with ASTSMC and fixed gain STSMC controllers in XY plane. From the presented results, it is obvious that the proposed ASTSMC controllers ensure robust formation control between the leader and followers UAVs, while with fixed gain STSMC controllers, all UAVs show drift in their trajectories.

**Figure 9:**
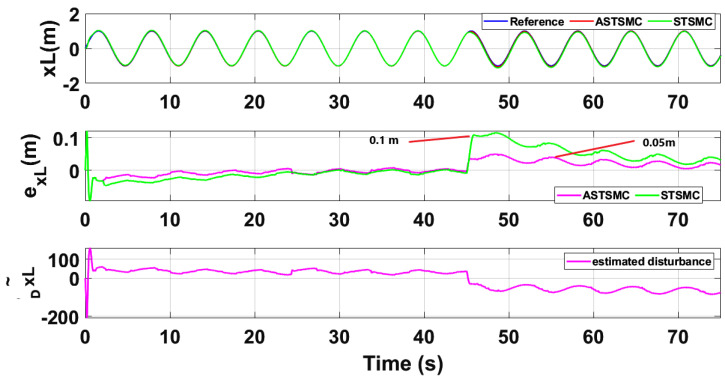
$X_{leader}$ tracking comparison under wind disturbance.

**Figure 10:**
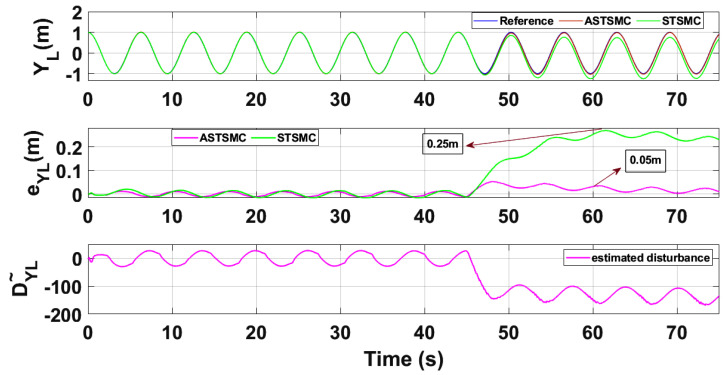
$Y_{leader}$ tracking comparison under wind disturbance.

**Figure 11:**
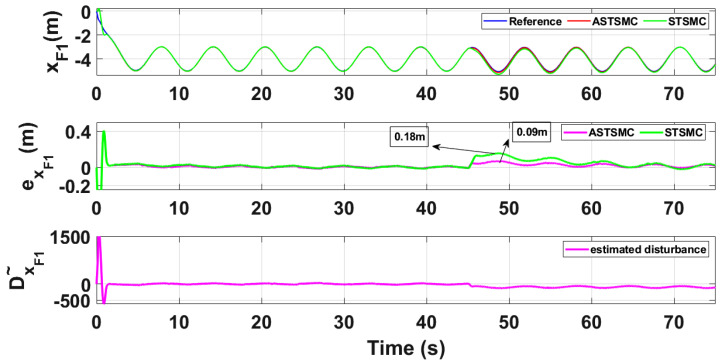
$X_{F1}$ tracking comparison under wind disturbance.

**Figure 12:**
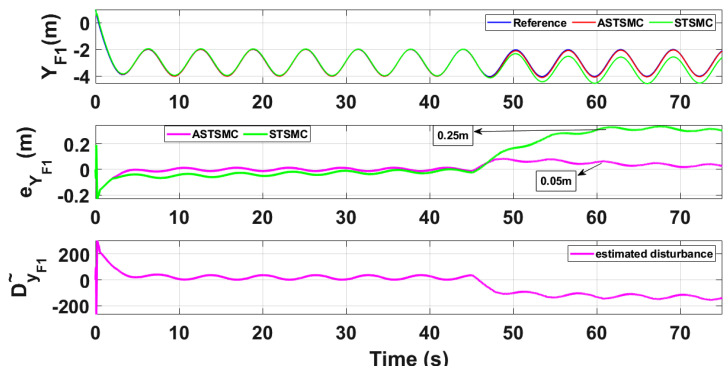
$Y_{F1}$ tracking comparison under wind disturbance.

**Figure 13:**
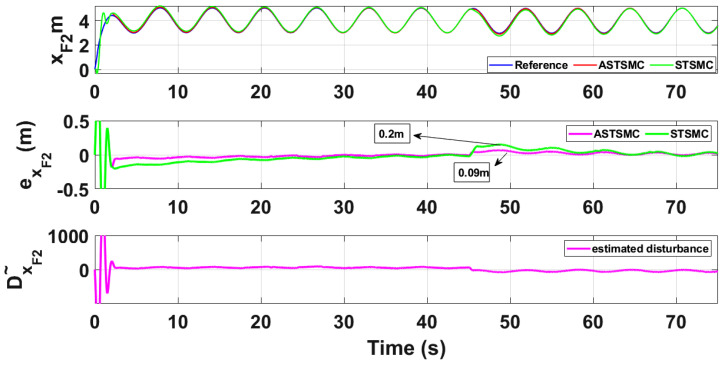
$X_{F2}$ tracking comparison under wind disturbance.

**Figure 14:**
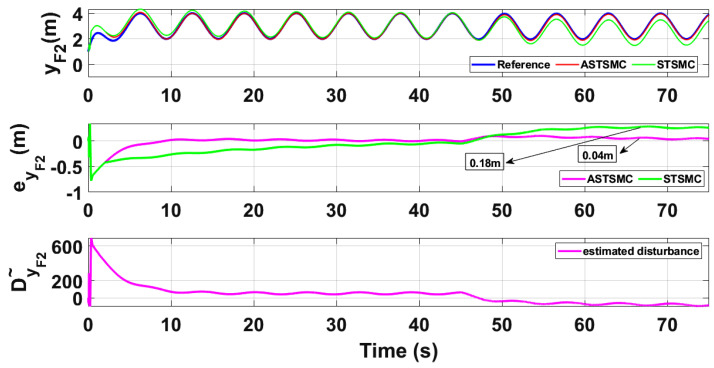
$Y_{F2}$ tracking comparison under wind disturbance.

To compare the trajectory tracking performance of the leader followers UAV quantitatively, the *X* and *Y* trajectories are individually plotted against time and the results are presented in Figure 9 and Figure 10 for the leader UAV. From Figure 9, and at time *t* = 45 s, the $e_{XL}$ tracking error is 0.1 m with fixed gain STSMC controller while with ASTSMC controller, the measured error $e_{XL}$ is 0.05. ASTSMC ensures lowest error due the adaptive disturbance compensation term $D_{XL}$ and from the presented results of Figure 9, it is obvious that at time *t* = 45 s, the adaptive term $D_{XL}$ adds appropriate compensation to cancel the disturbance and it switches from 50 to −100. Similarly from Figure 10, $e_{YL}$ is measured 0.28 m and 0.05 m with fixed gain STSMC and ASTSMC controllers respectively. ASTSMC controller offers lowest error due to the adaptive estimator term $D_{YL}$. From the presented results of Figure 10, it is obvious that at time *t* = 45 s, the adaptive term $D_{YL}$ adds appropriate compensation to cancel the disturbance and it switches from 0 to −150. Similarly for followers UAV, the *X* and *Y* tracking responses are plotted against simulation time and the results are shown in Figure 11, Figure 12, Figure 13 and Figure 14. From the presented results and at time *t* = 45 s, the measured error signals with fixed gain STSMC controller are as follows: $e_{X_{F1}}=0.2$ m, $e_{Y_{F1}}=0.3$ m, $e_{X_{F2}}=0.2$ m, $e_{Y_{F1}}=0.5$ m, while with ASTSMC controllers, the errors are measured as follows: $e_{X_{F1}}=0.1$ m, $e_{Y_{F1}}=0.1$ m, $e_{X_{F2}}=0.05$ m, $e_{Y_{F1}}=0.05$ m. From the presented results of Figure 11, Figure 12, Figure 13 and Figure 14, it is obvious that at time *t* = 45 s, the adaptive terms $D_{X_{F1}}$,$D_{Y_{F1}}$, $D_{X_{F2}}$,$D_{Y_{F2}}$ add appropriate compensation to cancel the disturbances.

**Figure 15:**
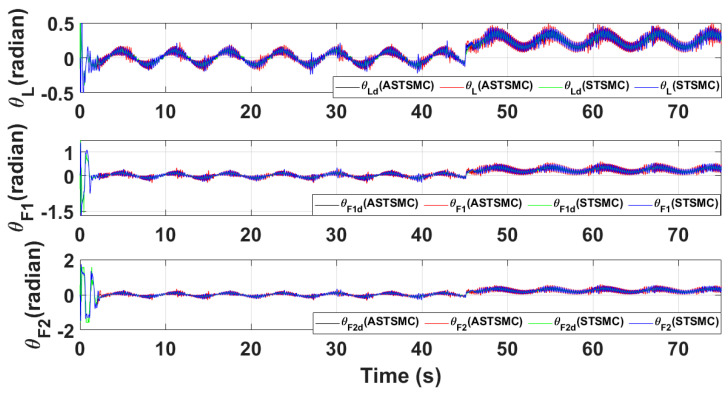
θ tracking comparison under wind disturbance.

**Figure 16:**
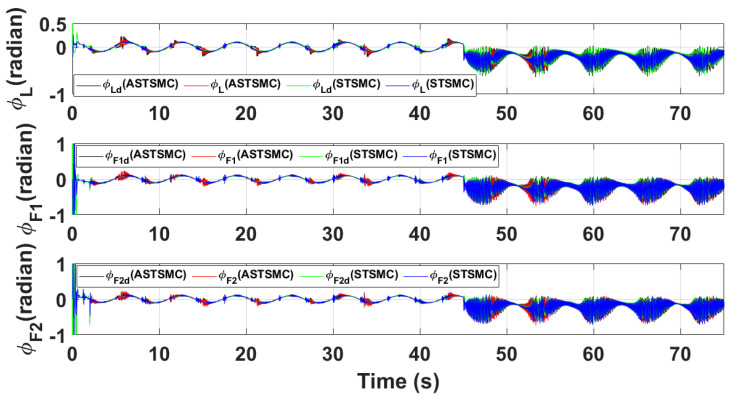
ϕ tracking comparison under wind disturbance.

**Figure 17:**
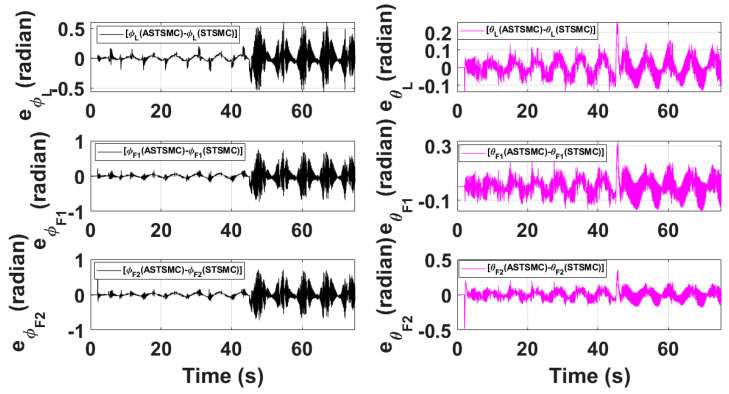
Difference between desired θ,ϕ with ASTSMC and STSMC controllers.

Figure 15 and Figure 16 show θ and ϕ tracking responses for leader and follower UAVs with both fixed gain STSMC and ASTSMC controllers respectively. From the presented results and at time *t* = 45 s, it is evident that the proposed ASTSMC controllers generate appropriate reference commands for both θ and ϕ loops of leader and follower UAVs. To have a better understanding of the above claim, Figure 17 shows the difference of the generated reference θ and ϕ commands with ASTSMC and fixed gain STSMC controllers. From the presented results, it is obvious that at time *t* = 45 s, the proposed ASTSMC controllers generate appropriate reference commands for both θ and ϕ loops of leader and follower UAVs.

**Figure 18:**
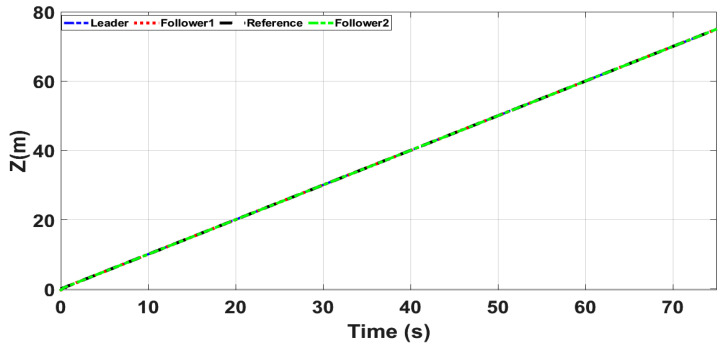
*z* tracking.

**Figure 19:**
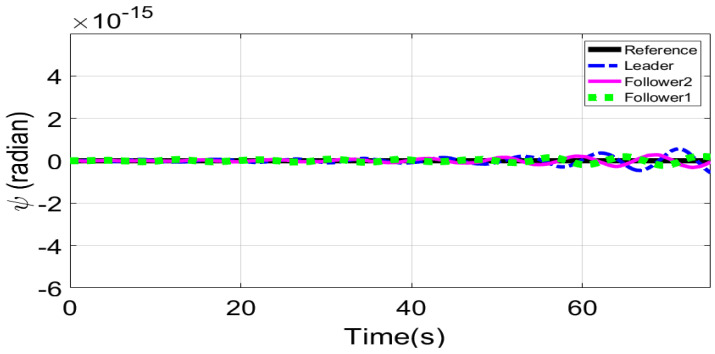
ψ tracking.

**Figure 20:**
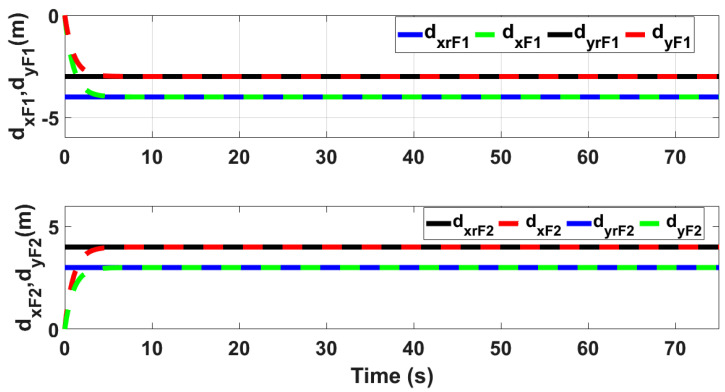
Formation controller tracking.

Figure 18 and Figure 19 show *Z* and ψ loops tracking responses for leader and follower UAVs. Since no disturbances are applied on both these loops, the tracking responses under fixed gain STSMC and ASTSMC controllers are comparable. Finally, Figure 20 shows the robustness of the formation controllers for tracking the respective reference commands i.e., the distance between the leader and the followers in *X*, *Y* plane. From the presented results it is obvious that apart from the transient error, the formation controllers accurately maintain the desired distance between the leader- follower1 and leader-follower2 UAVs.

**Figure 21:**
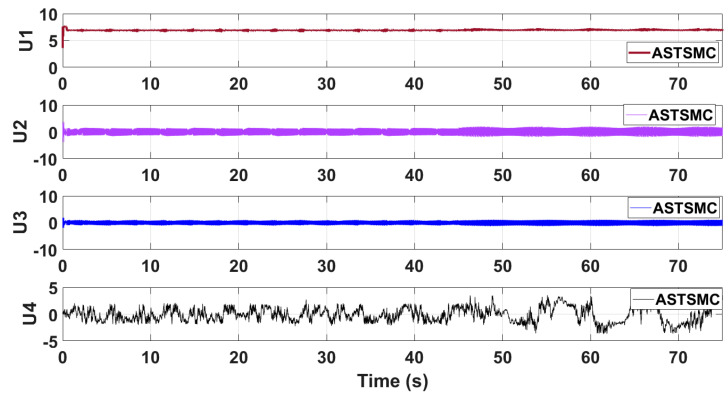
Control inputs using proposed control scheme.

Figure 21 shows the simulation results of the control inputs using the proposed control schemes. Since the attitude loops are not adaptive and gains are fixed so the control signals chatters, however the control inputs are feasible for practical implementations and well bounded. Moreover, the virtual XY control outputs of the proposed control schemes offer vert low chattering (Figure 15 and Figure 16) In future work, gains of the proposed control schemes will be tuned online to overcome the chattering phenomena.

## 5. Conclusions

This paper proposes adaptive super twisting sliding mode trajectory and formation controllers for multiple UAVs flying the leader follower configuration. Acceleration type disturbances and parametric uncertainties are applied to = *X* and *Y* dynamics of leader and follower UAVs. The formation control of UAVs is tested with the proposed ASTSMC and fixed gain STSMC controllers. The robust performance of the proposed control is verified from the following measured errors of the leader and follower UAVs. For leader UAV, $e_{XL}=0.05$ m, $e_{YL}=0.05$ m with ASTSMC control while with the fixed gain STSMC controller, the measured errors are as follows: $e_{XL}=0.1$ m, $e_{YL}=0.25$ m. Similarly for follower UAV,$e_{X_{F1}}=0.09$ m, $e_{Y_{F1}}=0.05$ m, $e_{X_{F2}}=0.09$ m, $e_{Y_{F1}}=0.04$ m, with ASTSMC control while with the fixed gain STSMC controller, the measured errors are as follows: $e_{X_{F1}}=0.18$ m, $e_{Y_{F1}}=0.25$ m, $e_{X_{F2}}=0.2$ m, $e_{Y_{F1}}=0.14$ m. Moreover, the settling time of XY states after the occurrence of disturbances is faster as compared to fixed gain STSMC control methods. From the quantitative comparison here; it is concluded that the proposed ASTSMC controllers show enhanced robust behaviour to the acceleration type disturbances and parametric uncertainties of the system. 

## Figures and Tables

**Table 1:** Leader-followers UAV parameters.

Symbol	Value	Unit
$m_{QL}=m_{F1}=m_{F1}$	0.65	kg
$l_{L}=l_{F1}=l_{F2}$	0.23	m
$J_{rL}=J_{F1}=J_{F2}$	$6.5\times 10^{-5}$	kg·m${}^{2}$
$I_{xL}=I_{xF1}=I_{xF2}$	$7.5\times 10^{-3}$	Ns${}^{2}$rad${}^{-1}$
$I_{yL}=I_{yF1}=I_{yF2}$	$7.5\times 10^{-3}$	Ns${}^{2}$rad${}^{-1}$
$I_{zL}=I_{zF1}=I_{zF2}$	$1.3\times 10^{-2}$	Ns${}^{2}$rad${}^{-1}$

**Table 2:** Leader UAV control parameters for attitude, altitude and position loops.

Parameter	Value	Parameter	Value
$k_{1}$	200	$k_{2}$	1
$k_{d1}$	70	$k_{d2}$	15
$k_{3}$	200	$k_{4}$	1
$k_{d3}$	50	$k_{d4}$	10
$k_{5}$	95	$k_{6}$	1
$k_{d5}$	4.6	$k_{d6}$	0.5
$k_{7}$	97	$k_{8}$	1
$k_{d7}$	300	$k_{d8}$	1.5
$k_{9}$	60	$k_{10}$	1000
$k_{d9}$	2.5	$k_{d10}$	180
$k_{11}$	60	$k_{12}$	1000
$k_{d11}$	2.5	$k_{d12}$	5
$\zeta_{1}$	1.5	$\zeta_{2}$	2.5

**Table 3:** Follower UAVs control parameters for attitude, altitude and position loops.

Parameter	Value	Parameter	Value
$k_{1j}$	200	$k_{2j}$	1
$k_{d1j}$	70	$k_{d2j}$	15
$k_{3j}$	200	$k_{4j}$	1
$k_{d3j}$	50	$k_{d4j}$	10
$k_{5j}$	95	$k_{6j}$	1
$k_{d5j}$	4.6	$k_{d6j}$	0.5
$k_{7j}$	97	$k_{8j}$	1
$k_{d7j}$	300	$k_{d8j}$	1.5
$k_{9j}$	60	$k_{10j}$	1000
$k_{d9j}$	2.5	$k_{d10j}$	180
$k_{11j}$	60	$k_{12j}$	1000
$k_{d11j}$	2.5	$k_{d12j}$	5
$\zeta_{1j}$	1.5	$\zeta_{2j}$	2.5

## Data Availability

Authors confirm the availability of all the supporting material and findings in the manuscript.

## Nomenclature

$J_{ri}$	Rotor inertia of $i^{th}$ quad-rotor [kg·m${}^{2}$]
*g*	Acceleration due to gravity [m/s${}^{2}$]
$D_{Xi}$	Uncertainty in $\ddot{X}$ dynamics of $i^{th}$ quad-rotor [m/s${}^{2}$]
$D_{Yi}$	Uncertainty in $\ddot{Y}$ dynamics of $i^{th}$ quad-rotor [m/s${}^{2}$]
$\Omega_{ri}$	Overall speed of the propellers of $i^{th}$ quad-rotor [rad/s]
$I_{xi,}I_{yi,}I_{zi}$	Moments of $i^{th}$ quad-rotor inertia in X, Y and Z coordinates [kg·m${}^{2}$]
$m_{Qi}$	Mass of $i^{th}$ quad-rotor [kg]
$M_{\phi }$ ${}_{i}$	Roll moment of $i^{th}$ quad-rotor [Nm]
$M_{\theta }$ ${}_{i}$	Pitch moment of $i^{th}$ quad-rotor [Nm ]
$M_{\psi }$ ${}_{i}$	Yaw moment of $i^{th}$ quad-rotor [Nm ]
$\theta_{i}$	Pitch angle of $i^{th}$ quad-rotor [rad]
$\phi_{i}$	Roll angle of $i^{th}$ quad-rotor [rad]
$\psi_{i}$	Yaw angle of $i^{th}$ quad-rotor [rad]
$X_{i},Y_{i},Z_{i}$	Position of $i^{th}$ quad-rotor in earth coordinates [m]
${\dot{X}}_{i},{\dot{Y}}_{i},{\dot{Z}}_{i}$	Velocity of $i^{th}$ quad-rotor in earth coordinates [m/s]
${\ddot{X}}_{i},{\ddot{Y}}_{i},{\ddot{Z}}_{i}$	Accelerations of $i^{th}$ quad-rotor in earth coordinates [m/s${}^{2}$]
